# GATA-1 Defects in Diamond–Blackfan Anemia: Phenotypic Characterization Points to a Specific Subset of Disease

**DOI:** 10.3390/genes13030447

**Published:** 2022-02-28

**Authors:** Birgit van Dooijeweert, Sima Kheradmand Kia, Niklas Dahl, Odile Fenneteau, Roos Leguit, Edward Nieuwenhuis, Wouter van Solinge, Richard van Wijk, Lydie Da Costa, Marije Bartels

**Affiliations:** 1 Central Diagnostic Laboratory Research, University Medical Center Utrecht, 3584 CX Utrecht, The Netherlands; b.vandooijeweert@umcutrecht.nl (B.v.D.); w.w.vansolinge@umcutrecht.nl (W.v.S.); r.vanwijk@umcutrecht.nl (R.v.W.); 2 Department of Pediatric Hematology, van Creveldkliniek, University Medical Center Utrecht, 3584 CX Utrecht, The Netherlands; 3 Laboratory for Red Blood Cell Diagnostics, Sanquin, 1006 AD Amsterdam, The Netherlands; simakia@gmail.com; 4 Peyvand Lab Complex, Shiraz 7363871347, Iran; 5 Department of Immunology, Genetics and Pathology, Uppsala University and Children’s Hospital, 751 85 Uppsala, Sweden; niklas.dahl@igp.uu.se; 6 AP-HP, Service d’Hématologie Biologique, Hôpital Robert Debré, University of Paris Cité, Hematim EA 4666, UPJV, F-75019 Paris, France; odile.fenneteau@aphp.fr (O.F.); lydie.dacosta@aphp.fr (L.D.C.); 7 Department of Pathology, University Medical Center Utrecht, 3584 CX Utrecht, The Netherlands; r.j.leguit-2@umcutrecht.nl; 8 Department of Pediatrics, University Medical Center Utrecht, 3508 AB Utrecht, The Netherlands; e.e.s.nieuwenhuis@umcutrecht.nl

**Keywords:** Diamond–Blackfan anemia, GATA-1, DBA-like disease, dyserythropoiesis, dysmegakaryopoiesis

## Abstract

Diamond–Blackfan anemia (DBA) is one of the inherited bone marrow failure syndromes marked by erythroid hypoplasia. Underlying variants in ribosomal protein (RP) genes account for 80% of cases, thereby classifying DBA as a ribosomopathy. In addition to RP genes, extremely rare variants in non-RP genes, including *GATA1*, the master transcription factor in erythropoiesis, have been reported in recent years in patients with a DBA-like phenotype. Subsequently, a pivotal role for GATA-1 in DBA pathophysiology was established by studies showing the impaired translation of *GATA1* mRNA downstream of the RP haploinsufficiency. Here, we report on a patient from the Dutch DBA registry, in which we found a novel hemizygous variant in *GATA1* (c.220+2T>C), and an Iranian patient with a previously reported variant in the initiation codon of *GATA1* (c.2T>C). Although clinical features were concordant with DBA, the bone marrow morphology in both patients was not typical for DBA, showing moderate erythropoietic activity with signs of dyserythropoiesis and dysmegakaryopoiesis. This motivated us to re-evaluate the clinical characteristics of previously reported cases, which resulted in the comprehensive characterization of 18 patients with an inherited GATA-1 defect in exon 2 that is presented in this case-series. In addition, we re-investigated the bone marrow aspirate of one of the previously published cases. Altogether, our observations suggest that DBA caused by *GATA1* defects is characterized by distinct phenotypic characteristics, including dyserythropoiesis and dysmegakaryopoiesis, and therefore represents a distinct phenotype within the DBA disease spectrum, which might need specific clinical management.

## 1. Introduction

**Figure 1:**
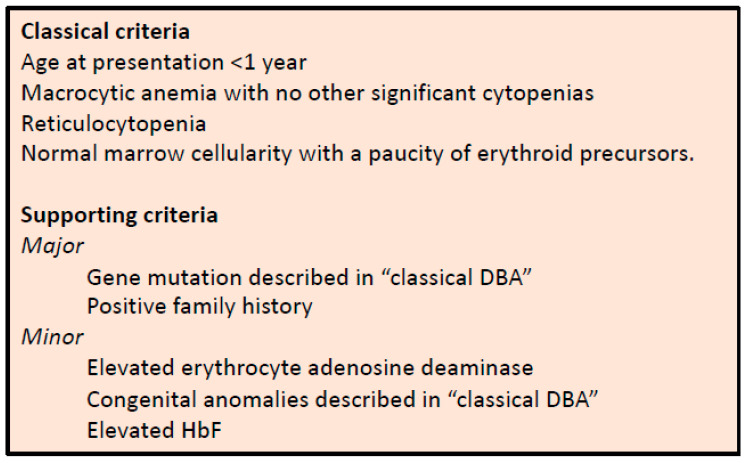
Diagnostic and supporting criteria for the diagnosis of DBA, as established by Vlachos et al. [3].

Diamond–Blackfan anemia (DBA) is a rare, inherited bone marrow failure syndrome that is characterized by hypoplastic anemia, congenital malformations (in ~50% of patients) and a predisposition to cancer [1,2]. The diagnosis of DBA is mainly based on clinical consensus criteria (Figure 1) [3]. Since the first groundbreaking study identifying *RPS19* variants as the underlying cause for DBA in 25% of cases [4], loss of function variants or deletions in more than 20 genes encoding ribosomal proteins (RPs) have been linked to DBA and can be identified in almost 75% of patients [5,6]. These variants cause ribosomal protein haploinsufficiency, leading to impaired ribosome biogenesis and ribosomal stress [7]. How the dysregulation of a basic cellular process such as ribosome biogenesis results in predominantly erythroid lineage-specific defects still remains enigmatic. Possible explanations proposed over the years include the hypersensitivity of erythroblasts to elevated p53 levels and a heme-versus globin-synthesis imbalance [8,9,10,11,12,13,14]. Just recently, it was recognized that p53 activation during ribosome biogenesis itself regulates normal erythroid differentiation [15]. Interestingly, in addition to RP genes, extremely rare variants in non-ribosomal genes such as *GATA1* have been linked to DBA-like diseases [16,17,18,19]. This is supported by recent and robust evidence of the impaired translation of GATA1 mRNA downstream of the RP haploinsufficiency, thereby linking these processes and providing an additional explanation for the selective erythroid defect in DBA [19,20,21].

GATA-1 is regarded as the “master regulator” of erythropoiesis, regulating processes in both erythroid differentiation and maturation. The protein itself contains two zinc finger domains and an N-terminal transactivation domain. An alternative initiation of translation results in the production of the full-length protein (GATA-1L) and a shorter isoform (GATA-1s) that lacks the N-terminal transactivation domain. GATA-1s is functionally incapable of supporting erythropoiesis adequately as a result of less (efficient) binding of GATA-1s to selective sites within erythroid target genes [22,23,24]. In addition to DBA-like diseases [16,17,18,19], variants in *GATA1* have been described in patients with X-linked thrombocytopenia with dyserythropoietic anemia (XLTDA), thrombocytopenia [25,26,27], X-linked (macro)thrombocytopenia with or without severe anemia (XLT) [28,29,30], X-linked thrombocytopenia with ß-thalassemia [31], macrocytic anemia and neutropenia [32], congenital erythropoietic porphyria (CEP) [33], as well as in acquired transient myeloproliferative disorder (TMD) and acute megakaryoblastic leukemia (AMKL) associated with Down syndrome [34,35,36,37]. *GATA1* variants associated with a DBA-phenotype all occur within the splice donor site of exon 2, resulting in the impaired production of full-length GATA-1 and the favored production of GATA1s [16,17,18,19].

In this study, we characterized two DBA patients in which *GATA1* defects in exon 2 were identified and retrospectively studied the clinical characteristics of previously reported patients with GATA-1 defects and DBA-like phenotypes. Together, our observations suggest that DBA caused by GATA-1 defects constitutes a distinct hematological and clinical phenotype within the DBA syndrome (DBA and DBA-like disease) [38], requiring specific aspects during clinical follow-up.

## 2. Methods

### 2.1. Patients and Genetics

The first patient was a patient from the Dutch DBA registry. The coding regions and flanking splice sites of 9 established DBA genes (*RPS7*, *RPS10*, *RPS17*, *RPS19*, *RPS24*, *RPS26*, *RPL5*, *RPL11* and *RPL35A)*, as well as *GATA1*, were assessed by Sanger sequencing. We found a novel hemizygous variant in *GATA1* (c.220+2T>C; p.?)), which was predicted to be pathogenic by in silico analysis (Supplementary File S1). The second patient was an Iranian patient who was identified at the age of five years old with severe anemia and treated at Namazi Hospital, Shiraz, Iran. At the age of thirteen, a next-generation sequencing (NGS) panel for RBC enzyme and membrane genes and targeted NGS panels for bone marrow failure, AML and MDS, were performed in The Netherlands (Sanquin, Amsterdam, The Netherlands), revealing a hemizygous variant in the initiation codon of *GATA1* (c.2T>C; p.?).

### 2.2. Literature Search

A retrospective literature analysis was conducted for all case reports and/or case series regarding inherited *GATA1* exon 2 defects in patients with hereditary anemia. The search strategy was therefore broad and comprised the search terms “*GATA1*” or “GATA-1” and “anemia”. Medline (Pubmed) was searched repeatedly between January 2020 and December 2021. The title and abstract (if provided) were screened, after which, the full text of potentially eligible studies was read. In addition, the reference lists of all the included articles were screened to search for additional records. Subsequently, studies that described patients with variants in exon 2 of *GATA1* were included. Data were extracted from the respective papers and their Supplemental Files.

### 2.3. Bone Marrow Analysis

Bone marrow aspirates and trephine biopsies were performed according to standard procedures and were analyzed by two independent cytologists (aspirates) and pathologists (biopsies), of which the second cytologist/pathologist was blinded from the clinical diagnosis.

### 2.4. Ethics

Written informed consent was obtained, and clinical and genetic data for our institutional Dutch and Iranian patients were retrieved from their electronic patient records and/or treating physicians.

## 3. Results

### 3.1. Hematological Characteristics of Two Novel DBA-Like Patients with GATA1 Defects

**Figure 2:**
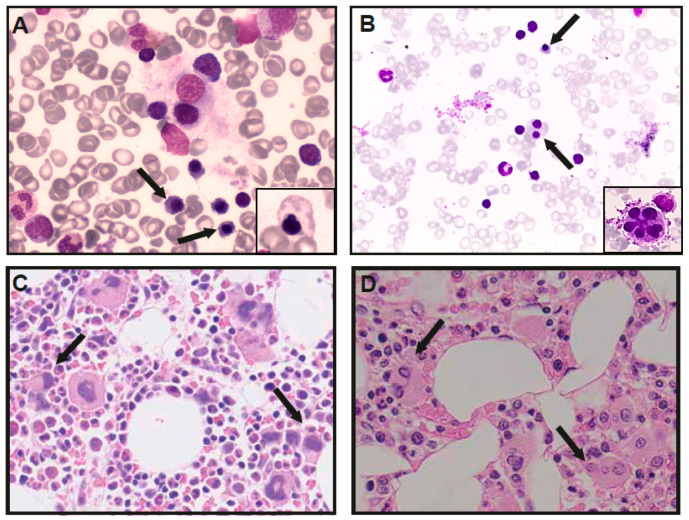
Bone marrow analysis of GATA-1 DBA patients. Bone marrow aspirates demonstrating dysplastic erythroid precursor cells (arrows), and aberrant shapes (small panel) in patient 1 (**A**) and dyserytropoiesis (arrows) and dysplastic megakaryocytes (small panel) in patient V-I (**B**). Trephine biopsies, demonstrating normocellular bone marrow with increased, dysplastic megakaryopoiesis (arrows) in patient 1 (**C**) and reduced cellularity and erythropoiesis with dysmegakaryopoiesis in patient V-I (**D**).

In the Dutch DBA registry, a male patient was identified with a novel *de novo* GATA1 c.220+2T>C splice site variant, which was predicted to cause the skipping of exon 2 and thus produce predominantly GATA-1s protein. The patient was an eleven-year-old boy of Somalian descent that presented in his native country with anemia at the age of seven months old. A bone marrow examination was performed and allegedly reported erythroid hypoplasia. He was treated in both Somalia and Ethiopia with regular blood transfusions before, and after he moved to The Netherlands at the age of four, he was started on glucocorticoid treatment. At that point, his CBC and additional investigations showed mild macrocytic anemia (Hb 9.4 g/dL, MCV 100 fL, references Hb 11.5–14.0 g/dL, MCV 75–85 fL) with a normal reticulocyte count, normal neutrophil and platelet counts, increased fetal hemoglobin (HbF 3.3%) and normal erythrocyte adenosine deaminase activity (eADA 1.0 U/g Hb). No congenital malformations were found, whereas length growth was below the target height range (−2.5 SD). During the follow-up, and while on treatment with glucocorticoids (0.2 mg/kg/day), two bone marrow examinations were performed, demonstrating no significant erythroid hypoplasia (an absence of reticulocytopenia) yet significant dyserythropoiesis and mild dysmegakaryopoiesis (Figure 2). Until the last follow-up, he had a stable disease with glucocorticoid treatment (0.2 mg/kg/day), characterized by moderately severe anemia (Hb 8.7–9.4 g/dL, reference 11.5–15.5 g/dL) with normal reticulocyte counts (70–100 × 10^9^/L), a short stature but a normal growth velocity (at −2.5 SD) and no physical complaints.

The second patient was an Iranian boy who had been treated since the age of five years old for severe macrocytic anemia at Shiraz Medical University hospital (Hb 8.2 g/dL, MCV 97 fL, references Hb 11.5–14.0 g/dL, MCV 75–85 fL). A bone marrow aspirate performed at the age of six years old showed normal cellularity and erythropoiesis (27% erythroblast; reference 8–30%), with megaloblastic changes with no significant dysplastic features (morphology not available). After the first attempt failed, the patient is currently being treated with glucocorticoids (Hb 8.5–10.2 g/dL). Follow-up blood counts often showed increased platelet counts (mean 652 × 10^9^/L), and no congenital malformations were noted. At the age of thirteen, a molecular analysis was performed in The Netherlands, demonstrating the previously reported *GATA1* (c.2T>C) variant [18].

### 3.2. Comparative Analysis with Previously Reported Cases Illustrates Distinct DBA-Like Disease Characteristics

**Figure 3:**
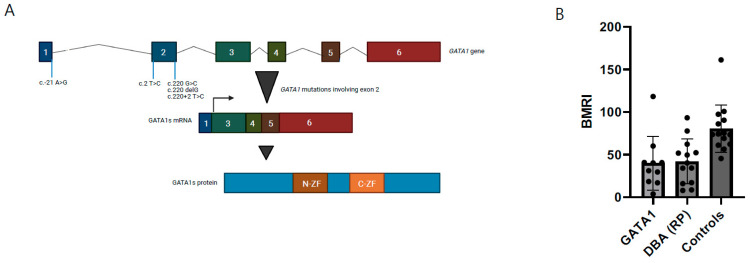
Functional characteristics of GATA-1 DBA. (**A**) Overview of genetic defects at exon 2 in *GATA1*, inducing GATA1s exclusively (figure adapted from Ling & Crispino) [40]. (**B**) Bone marrow responsiveness index (BMRI), calculated as [(absolute reticulocyte count) × (patient’s Hb/normal Hb)], depicted for DBA patients with *GATA1* defects, ribosomal protein defects from the Dutch DBA Registry and healthy controls.

In addition to our two novel patients, sixteen patients from six families were identified in our PubMed literature search [16,17,18,19,32]. All patients were males and had variants in either exon 2 or the start codon, in both cases leading to the predominant/exclusive synthesis of the short isoform of GATA1 (GATA1s) (Table 1). For all patients, data were available on the age of onset, demonstrating that ten patients (10/18, 56%) were diagnosed before the age of one year old. Thirteen patients (13/18, 72%) presented with macrocytic anemia. Regarding other hematopoietic lineages, seven patients (7/18, 39%) had a leukocyte count below the normal range and mild to severe neutropenia. Absolute reticulocytopenia was present in four patients in which data were available (4/14, 29%). However, when a “bone marrow responsiveness index” (BMRI) was calculated, a measure to consider the reticulocyte count relative to the degree of anemia, eight additional patients proved to have relative reticulocytopenia (85.7%), comparable to DBA based on RP-gene defects (Figure 3) [39]. So far, no malignancies have been reported in the GATA1 mutated patients, except for the one with MDS [18]. However, we cannot eliminate an accidental association in this small number of patients.

From fifteen patients (including this report), bone marrow characteristics were reported, demonstrating normocellular bone marrow with a paucity of erythroid precursors in six patients (6/15, 40%). One of these patients (Index XIII) was diagnosed with myelodysplastic syndrome (MDS) by the age of four. (Moderate) hypocellularity was noted in six patients (6/15, 40%), and in one patient, moderate hypercellularity was found (index VI-I). Dysplastic features were reported in 9/15 (60%) patients and in 7/15 (47%) patients in the erythroid lineage specifically (dyserythropoiesis). In addition, we re-analyzed the bone marrow aspirate of patient V-I, which had been described as “erythroid hypoplasia with otherwise normal cellularity”. Revision by two blinded cytologists illustrated dysplastic erythropoiesis with dysmorphic cells and dysmegakaryopoiesis (micromegakaryocytes and fragmented megakaryocytes), which had also been reported in patients from family I (Table 1, Figure 1C,D). Patient V-I was not treated with glucocorticoids at the time of the bone marrow analysis. Nine (9/18, 50%) patients treated with glucocorticoids had an initial or partial treatment response, yet four patients (4/9, 44%) became transfusion-dependent after cessation of effect. Three patients were treated with a hematopoietic stem cell transplantation (HSCT), which cured their hematological disease. Indications for HSCT were severe combined anemia and neutropenia (Index I-IV and V) and MDS (Index IV-I). Seven patients are on regular erythrocyte transfusions (7/18, 38%), three patients receive no treatment (17%) and two patients died (2/18, 11%), reportedly from severe pneumonia (Index II) and an unrelated cause (index VIII).

In summary, this suggests that DBA based on specific *GATA1* defects is characterized by macrocytic anemia, with neutropenia in a significant number of patients, and specific bone marrow cytomorphological abnormalities, including dyserythropoiesis, dysmegakaryopoiesis with micromegakaryocytes and fragmented megakaryocytes. In addition, other specific characteristics of DBA, including reticulocytopenia, congenital malformations and elevated eADA, are missing. Fetal Hb (HbF) was elevated in all of the analyzed patients. The distribution of patients between treatment modalities is in line with the whole DBA population.

## 4. Discussion

In this study, we reported on two new DBA-like patients with variants in exon 2 of the erythroid transcription factor GATA-1 and reviewed all published cases. Our subsequent comprehensive review of the cases illustrates that inherited *GATA1* defects in exon 2 represent a hematological phenotype that displays distinct characteristics within the DBA spectrum, including (severe) neutropenia and dyserythropoiesis with or without dysmegakaryopoiesis, instead of the typical quantitative intrinsic erythroid defect (hypoplastic anemia). GATA-1 is considered the master transcription factor of erythropoiesis and is known to regulate processes in both early and late erythroid development. In addition to erythroid cells, GATA-1 is expressed in megakaryocytes, eosinophils and basophils, yet is not expressed in non-hematological tissues [41]. In mouse models, the knockdown of *Gata1* leads to maturation arrest at the proerythroblast stage, thrombocytopenia and the increased proliferation of megakaryocytes, whereas *Gata1*-null mice are embryonically lethal due to severe anemia [42,43]. The GATA-1 protein has three important functional domains for erythropoiesis: the N-terminal transactivation domain (N-TAD), an N-terminal zinc finger domain (N-ZF) and a C-terminal zinc finger domain (C-ZF) [40]. The short isoform of GATA-1, GATA-1s, starts at a methionine at position 37 and does not include the N-TAD, and therefore cannot support erythropoiesis adequately. *GATA1* variants have been associated with a variety of hematological phenotypes, which can be largely explained by their implication for GATA-1 protein function and GATA-1 binding to FOG-1 [22,40]. GATA-1 also directly or indirectly regulates the development of other hematopoietic lineages, including megakaryopoiesis and granulopoiesis, through interaction with the myeloid master transcription factor PU.1 and by the downregulation of GATA-2 expression [22,40,44], which could explain the abnormalities in megakaryocyte and platelet numbers in patients. Within the spectrum of GATA-1 red cell disorders, the DBA-like phenotype is associated with variants at exon 2 boundaries, resulting in a favored production of the short isoform at the expense of the FL GATA-1 protein. Since it was demonstrated that *GATA1* translation is impaired downstream of the RP haploinsufficiency in DBA, this provides an additional explanation for the selective erythroid defect in the case of RP haploinsufficiency, and the clinical characteristics of DBA patients with GATA-1 defects [20,21]. However, it could also point out the distinct phenotypic features, illustrated by an exclusively hematological phenotype in GATA-1 DBA-like patients, compared with a multiorgan disease, encompassing congenital malformations and an increased risk to also develop solid tumors (in addition to hematological malignancies) in the case of DBA with genetic defects in genes encoding RP. While the number of patients studied is too small to make a conclusive statement, solid tumors have not been diagnosed in all GATA-1 DBA-like patients so far. In one patient, MDS evolution has been identified without ruling out an accidental event not related to the *GATA1* gene variant. Importantly, some RP genes (e.g., *RPL5*, *RPL11* and *RPS20*) are known to be tumor-suppressor genes, which is not the case for *GATA1*. Theoretically, this could play a role in the absence of malignancies, in particular solid tumors in GATA-1 DBA-like patients [45,46,47].

Our retrospective, comprehensive characterization illustrates that the vast majority of patients do not meet the widely used diagnostic criteria for classical DBA, illustrated by the absence of reticulocytopenia in ten analyzed patients, neutropenia in seven (39%) patients and no typical bone marrow morphological findings [3]. Although leukocyte abnormalities are not rare in DBA related to RP defects, these predominantly encompass lymphocytes and lymphocyte subsets (NK-cells, T-, and B-lymphocytes), whereas neutropenia in DBA is associated with *RPL35a* defects [48,49]. In addition, no congenital malformations were described, and increased HbF is arguably not specific for DBA (minor criteria), yet it largely increased in GATA-1 DBA-like patients. Since eADA was only analyzed in six out of eighteen patients, this cannot be compared with DBA patients with RP defects, in which eADA is elevated in the majority (>80%) of patients [50]. Of special interest in GATA-1 DBA-like patients are their bone marrow cytomorphology findings. Contrary to the classical hypoplastic anemia reported in DBA patients with RP defects, dysplastic features and dyserythropoiesis are reported in 60% and 47% of patients with GATA-1 defects, respectively. Dyserythropoiesis, as well as dysmegakaryopoiesis, was also present in the Dutch patient we reported on. In addition, a blinded revision of previously reported, typical DBA findings in the Swedish GATA-1 patient also showed clear dysplastic erythropoiesis in addition to dysmegakaryopoiesis (including micro- and fragmented megakaryocytes), a finding that was previously reported in the Brazilian family by Hollanda et al [32]. These findings suggest that the bone marrow characteristics in GATA-1 DBA-like patients are distinct from the typical bone marrow findings in DBA with RP gene variants, characterized by a quantitative intrinsic erythroid defect or erythroblastopenia (absence or less than 5% of erythroblasts in otherwise normocellular bone marrow with no signs of dysplasia).

In conclusion, our observations suggest that DBA caused by *GATA1* defects is characterized by distinct phenotypic characteristics, including dyserythropoiesis, abnormal megakaryopoiesis and neutropenia, and therefore represents a distinct phenotype within the DBA disease spectrum, which might require specific clinical management.

## Figures and Tables

**Table 1:** Clinical and molecular characteristics of reported cases.

Index#	Reported	Molecular Defect	Type	Age Diagnosis	Hgb (g/dL)	HbF (%)	MCV (fL)	Retics (×10^9^/L)	WBC (×10^9^/L)	ANC (×10^9^/L)	PLts (×10^9^/L)	eADA	Steroid Responsive	Current Treatment	Bone Marrow
I-I	Hollanda et al. 2006	c.220G>C	missense	40 years	10.9	NA	100.6	NA	5.9	2.8	352	NA	NA	none	Moderate hypercellularity in 3 lineages. Moderate reduction in relationship between G/E. Retardation of maturation of the granulocytic and erythroblast series. Moderate number of micromegakaryocytes. Neutrophils with pseudo Pelger–Hüet anomaly.
I-II		c.220G>C	missense	24 years	7.7	NA	99.0	56	3.7	1.2	161	NA	NA	deceased (severe pneumonia)	Hypocellularity with normal number of megakaryocytes and frequent micromegakaryocytes.
I-III		c.220G>C	missense	35 years	11.8	NA	100.8	139	5.2	2.0	190	NA	NA	none	NA
I-IV		c.220G>C	missense	12 years	5.2	↑	94	108	2	0.6	135	NA	NA	remission after allogeneic BMT	Moderate hypocellularity with multinucleation and nuclear karyorrhexis in erythroblasts. Neutrophils with pseudo Pelger–Hüet anomaly and moderate number of micromegakaryocytes. No ringed sideroblasts were seen.
I-V		c.220G>C	missense	20 years	3.8	↑	101	108	1.6	0.5	144	NA	NA	remission after allogeneic BMT	Moderate hypocellularity with multinucleation and nuclear karyorrhexis in erythroblasts. Neutrophils with pseudo Pelger–Hüet anomaly and moderate number of micromegakaryocytes. No ringed sideroblasts were seen.
I-VI		c.220G>C	missense	2 months	6.1	NA	93	NA	7.5	NP	345	NA	NA	regular transfusions	Normocellularity with moderate reduction in the relationship between G/E. Maturation preserved and moderate number of megakaryocytes, with micromegakaryocytes.
I-VII		c.220G>C	missense	4 years	5.3	↑	88.1	44.2	2.2	0.6	294	NA	NA	regular transfusions	Moderate hypocellularity with multinucleation and nuclear karyorrhexis in erythroblasts. Neutrophils with pseudo Pelger–Hüet anomaly and moderate number of micromegakaryocytes. No ringed sideroblasts were seen.
I-VIII		c.220G>C	missense	17 years	9.6	↑	102.8	58.1	3.7	1	400	NA	NA	deceased (‘unrelated cause’)	Moderate hypocellularity with multinucleation and nuclear karyorrhexis in erythroblasts. Neutrophils with pseudo Pelger–Hüet anomaly and moderate number of micromegakaryocytes. No ringed sideroblasts were seen.
II-I	Sankaran et al. 2012	c.220G>C	missense	birth	(lab 23y) 8.4	↑	NA	21	3.1	0.83	71	normal	YES (temporarily)	regular transfusions	Erythroid hypoplasia without abnormalities of the other hematopoietic lineages.
II-II		c.220G>C	missense	1.6 months	(lab 19y) 8.3	NA	100	68	4.5	2.1	201	NA	YES (temporarily)	regular transfusions	Erythroid hypoplasia without abnormalities of the other hematopoietic lineages.
II-III		c.220delG	frameshift	6 weeks	3.5	↑ *	not reported	unknown	6.8	1.6 (?)	362 (?)	normal	YES	glucocorticoids	Not documented.
III-I	Ludwig et al. 2014	c.2T>C		not reported	9.7	NA	101.8	73.2	6.2	3.9	239	NP	NA	regular transfusions	Not documented.
IV-I	Parrella et al. 2014	c.2T>C		9 months	5.5	NA	93	40	normal range	normal range	normal range	↑	partial response	remission after allogeneic BMT	Selective deficiency in erythroid precursors without abnormalities in the other hematopoietic lineages --> 4 yrs: severe hypocellular bone marrow with a 45XY, −7 clone (65%) and a further 50XXY, +3, +8, +21 clone (MDS).
V-I	Klar et al. 2014	c.220G>C	missense	<3 months	5.0–9.0	↑	104–108	“low”	normal range	normal range	normal range	↑	YES (temporarily)	regular transfusions	Erythroid hypoplasia with otherwise normal cellularity.
V-II		c.220G>C	missense	<3 months	5.0–9.0	↑	104–108	“low”	normal range	normal range	normal range	↑	YES (temporarily)	regular transfusions	Erythroid hypoplasia with otherwise normal cellularity.
V-III		c.220G>C	missense	<3 months	5.0–9.0	↑	104–108	“low”	normal range	normal range	normal range	unknown	NA	none	Erythroid hypoplasia with otherwise normal cellularity.
VI-I	this paper, patient 1	c.220+2T>C	missense	7 months	9.5	↑	100	75.4	5.5	4.1	525	normal	YES	glucocorticoids	Mildly decreased erthropoiesis, dyserythropoiesis, increased megakaryopoiesis.
VII-I	this paper, patient 2	c.2T>C	missense	5 years	8.2	↑	97	NA	4.8	3.1	652	NA	YES,(partial)	glucocorticoids	Normal cellularity and erythroid activity with megaloblastic changes, no significant dysplastic features.

Clinical and molecular characteristics of all reported cases, including the patients presented in this paper, with variants in either exon 2 or the start codon leading to predominant/exclusive synthesis of the short isoform of GATA-1 (GATA-1s). Pink marking = higher than normal reference values, blue marking = lower than normal reference values. Reference values according to *Williams Hematology*, *10e* Eds. Kenneth Kaushansky et al. McGraw Hill, 2021. * Normal for young age.

## Supplementary Materials

The following supporting information can be downloaded at: https://www.mdpi.com/article/10.3390/genes13030447/s1, Supplementary Information S1: In silico predictions and *GATA1* variant electropherogram.
