# Supplementary material for: GATA-1 Defects in Diamond–Blackfan Anemia: Phenotypic Characterization Points to a Specific Subset of Disease
Source: Genes (Basel). 2022 Feb 28;13(3):447. doi: 10.3390/genes13030447 (PMC8949872; doi:10.3390/genes13030447)

Supplementary File S1. In silico predictions

GATA1 gene (Xq11.23):

NM_002049.4

Patient 1.
***GATA1* c.220+2T>C; p.?**
intron 2
Splicing site mutation
Position 48791331
gnomAD: 0
Class 5 pathogenic according to ACMG-AMP (Richards et al., Genet Med 2015 ; Amendola et al., Am J Hum Genet 2016).
No publications about this variation; no metascore; no clinVar description
CADD= 26.4
PHRED=26.4
Rawscore: 3.91
Varsome data:
Individual predictions: bayesDel addAF=damaging; bayesDel noAF= damaging
FATHMM-MKL= damaging
Mutation taster disease causing

Patient 2.
***GATA1* c.2T>C; p.(Met1Thr)**
exon 2
start loss allelic variation
position 48791111
rs587776451
gnomAD=0
Class 5 pathogenic according to ACMG-AMP (Richards et al., Genet Med 2015 ; Amendola et al., Am J Hum Genet 2016).
4 publications: Bluteau et al, Blood; Lutwig LS et al., Nature med 2014; Parrella et al., S J Pediatr Blood and Cancer; Kanezaki et al., Blood
Clinvar: pathogenic RCV001382885, RCV000144252, RCV000754842
CADD=21.9
PHRED=21.9
Rawscore=2.35
Metascore: 4/4:
METALR=0.8322 damaging;
METASVM=0.5822 Damaging;
METARNN=0.8873 Damaging;
Revel=0.56 pathogenic
Individual predictions = 11/12 damaging
Bayesdel addAF score 0.7434 damaging;
Bayesdel noAF score 0.8299 damaging;
mutation taster disease causing;
sift/sift4G score 0 damaging

Electropherogram of ***GATA1* c.220+2T>C; p.?**

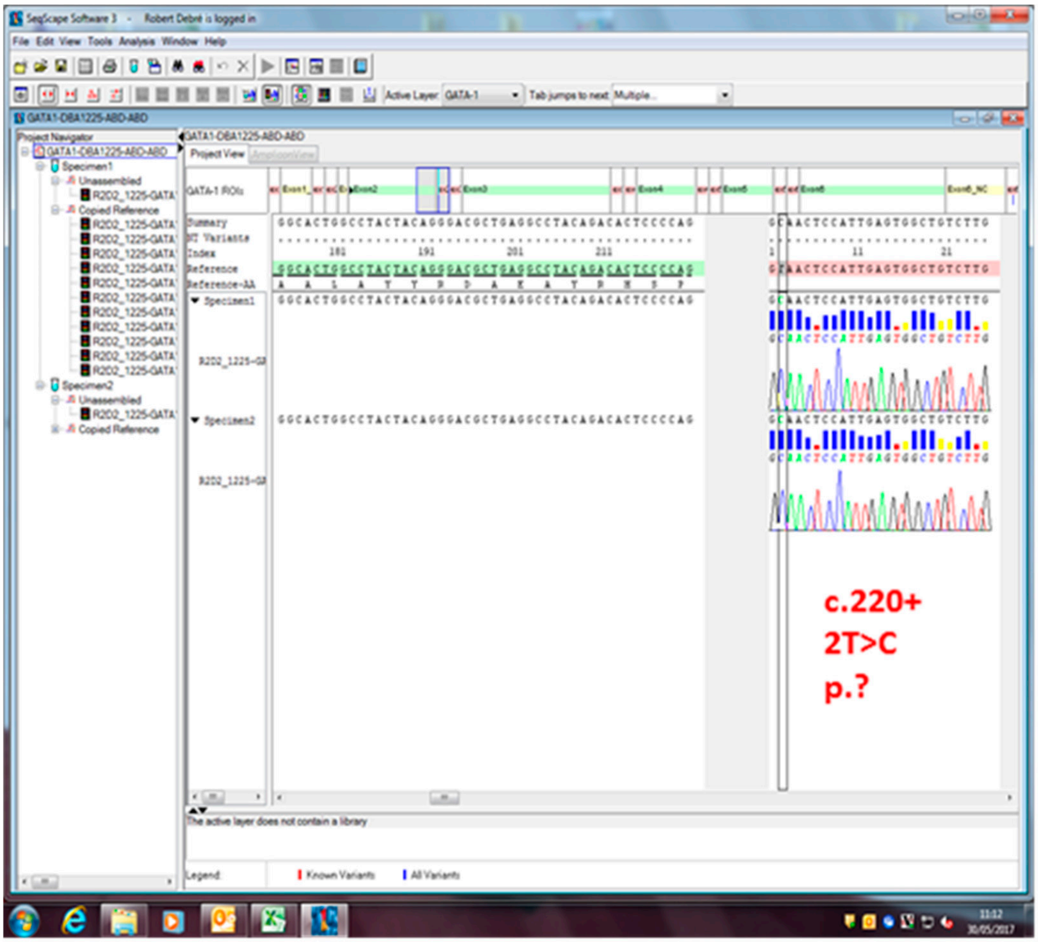

Supplement: Supplementary file 1 [file genes-13-00447-s001.zip › genes-1570525-supplementary.pdf]
